# Identifying the essential elements to inform the development of a research agenda for Paramedicine in Ireland: a Delphi Study

**DOI:** 10.1186/s12961-024-01188-6

**Published:** 2024-08-09

**Authors:** Kelly-Ann Bowles, Alan M. Batt, Michelle O’Toole, Shane Knox, Liam Hemingway, Julia Williams, Brett Williams, Niamh M. Cummins

**Affiliations:** 1 Department of Paramedicine, Monash University, Melbourne, Australia; 2 Faculty of Health Sciences, Queen’s University, Kingston, ON Canada; 3 RCSI SIM, Centre for Simulation Education and Research, RCSI University of Medicine and Health Sciences, Dublin, Ireland; 4 Irish Paramedicine Education and Research Network (IPERN), Limerick, Ireland; 5 National Ambulance Service, Dublin, Ireland; 6 School of Medicine, University College Cork, Cork, Ireland; 7 School of Health and Social Work, University of Hertfordshire, Hatfield, United Kingdom; 8 School of Medicine, SLÁINTE Research and Education Alliance in General Practice, Primary Healthcare and Public Health, Faculty of Education and Health Sciences, University of Limerick, Limerick, Ireland; 9 Ageing Research Centre, Health Research Institute, University of Limerick, Limerick, Ireland

**Keywords:** Paramedicine, Research priorities, Delphi, Ireland

## Abstract

**Background:**

Paramedicine is a dynamic profession which has evolved from a “treat and transport” service into a complex network of health professionals working in a diverse range of clinical roles. Research is challenging in the paramedicine context, and internationally, research capacity and culture has developed slowly. International examples of research agendas and strategies in paramedicine exist, however, research priorities have not previously been identified in Ireland.

**Methods:**

This study was a three round electronic modified Delphi design which aimed to establish the key aspects of the research priorities via end-user consensus. Participants included interested stakeholders involved in prehospital care or research in Ireland. The first round questionnaire consisted of open-ended questions with results coded and developed into themes for the closed-ended questions used in the second and third round questionnaires. A consensus level of 70% was set a priori for second and third rounds.

**Results:**

Research Priorities that reached consensus included Staff Wellbeing, Education and Professionalism and Acute Medical Conditions. Respondents indicated that these three areas should be a priority in the next 2 years. Education, Staffing and Leadership were imperative Key Resources that required change. Education was a Key Processes change deemed imperative to allow the future research to occur. Outcomes that should be included in the future research strategy were Patient Outcomes, Practitioner Development, Practitioner Wellbeing, Alternate Pathways, Evidence-based Practice and Staff Satisfaction.

**Conclusion:**

The results of this study are similar to previously published international studies, with some key differences. There was a greater emphasis on Education and Practitioner Wellbeing with the latter possibly attributed to the timing of the research in relation to the COVID-19 pandemic. The disseminated findings of this study should inform sustainable funding models to aid the development of paramedicine research in Ireland.

**Supplementary Information:**

The online version contains supplementary material available at 10.1186/s12961-024-01188-6.

## Background

Paramedicine is a dynamic profession which has evolved from a “treat and transport” ambulance service into a complex network of health professionals working in a diverse range of clinical roles. Research is particularly challenging in the unpredictable paramedicine contexts and therefore research capacity and culture has developed slowly internationally. Historically paramedicine has relied on evidence generated by other health professions or on “heirloom knowledge” developed through pragmatism. Education in paramedicine has also traditionally focused on vocational in-service training meaning there has been less emphasis on research skills. Therefore, while progress has been made in paramedicine research internationally, it must be acknowledged that a research gap remains across jurisdictions, regardless of the level of sophistication of the system or level of professional practice [1].

Irish paramedicine education and research has progressed significantly over the past two decades. In 2000 the establishment of a statutory regulator, the Pre-Hospital Emergency Care Council (PHECC) marked an important milestone in the professionalisation of Irish pre-hospital practitioners. A National Pre-hospital Research Strategy was published in 2008 [2] which was highly innovative at the time. A key milestone in education was achieved with the transition to Higher Education and the graduation of the first degree-educated paramedics in 2016. The foundation of the Irish Paramedicine Education and Research Network (IPERN) in 2021 was a further positive development for building research capacity and culture in the profession.

Building such capacity in the system is a priority across several key publications. First, the Health Service Executive (HSE) Action Plan for Health Research (2019–2029) in Ireland promotes embedding a culture of research, Evidence Based Practice (EBP) and innovation in the health service [3]. It aims to develop the mechanisms and operational infrastructure required to embed research as an integral part of health service delivery and decision-making. In addition, the Irish Association of Emergency Medicine (IAEM) promotes knowledge translation aimed at bridging the evidence-to-practice gap and improving clinical outcomes of patients in the emergency care setting [4]. Finally, in relation to prehospital and out-of-hospital research, the PHECC Strategic Plan 2020 highlights “Focused Research” as a key goal, with an emphasis on developing research capability and capacity [5]. The recent establishment of a Research Committee by PHECC is one development contributing to a unity of purpose by key stakeholders, towards building a vibrant research ecosystem in Irish out-of-hospital care.

Prioritising research within this ecosystem based on gaps in our understanding of issues is essential to determine the importance and relevance of conducting research. Research strategies in paramedicine exist in a number of countries, [6–8] and priority-setting exercises were conducted at a national level in some jurisdictions [9–15]. Work is underway to establish a new Canadian EMS Research agenda, while others are conducting a study establishing paramedicine research priorities internationally. While previous research highlighted key performance indicators for pre-hospital emergency care in Ireland [16], research priorities for paramedicine have not yet been identified. This step is key to informing future research developments and healthcare strategies for stakeholders such as patients, clinicians, researchers, advocates, policy-makers and funding bodies.

The aim of this study is therefore to establish, via consensus, the future Research Priorities for paramedicine in Ireland, giving attention to the short, medium, and long-term priority areas of research. Secondary aims are to identify the Key Resources and Key Processes required to allow the research priorities to be met and to establish measurable Outcomes in future research strategies.

## Methods

### Study design

A three round electronic modified Delphi Study design was implemented. The study was conducted in line with the Conducting and REporting DElphi Studies (CREDES) checklist [17]. We defined our expert group broadly, as any person with a stake in prehospital or out-of-hospital research in Ireland. This was deemed the most appropriate approach to answer the project question, as end-user contribution was imperative to the success of a resultant research strategy. The electronic application of the study design was also determined as the most appropriate to allow participation from diverse geographical areas. An a priori level of consensus was set at 70% of the cohort selecting the same category/Likert level. Items that met consensus were not presented in future rounds of the survey, however all other items were re-presented to respondents.

### Participants

Participants were any person involved in prehospital care in Ireland including First Responders, Emergency Medical Technicians (EMT), Paramedics, Advanced Paramedics (AP), Nurses, Doctors, Allied Health Clinicians and “Other Roles” (including professionals working in management, education, and research). To ensure a diverse range of perspectives were represented, expressions of interest were sought via email to all those on the PHECC Research Mailing list with advertisements also shared via PHECC, the Irish Paramedicine Education and Research Network (IPERN) and Monash University Department of Paramedicine social media accounts. The advertisement was additionally shared with key stakeholders in the field. All who provided an expression of interest (which included an email address for further communication) were then sent a link to the first-round questionnaire. The same respondents were sent the link to the second-round questionnaire, regardless of whether they completed the first round. Only respondents who completed the second-round questionnaire and provided a valid email address were sent the third-round questionnaire.

### Questionnaires

The first-round questionnaire contained an initial section including demographic questions relating to gender, age, role, time in the career and geographic area of service. Four essential elements were then investigated including the Research Priorities, Key Resources, Key Processes and Outcomes. The following sections included open-ended questions relating to short, medium and long-term areas of Research Priorities as well as Key Resources and Key Processes required to facilitate these changes. The first-round questionnaire was open for 10 days from the 31st of October until the 9th of November 2022.

Data collected in the first-round questionnaire was coded and developed into themes for the subsequent rounds. The second-round questionnaire presented a summary of the key findings from the first round in the form of an infographic (Appendix 1). The second-round questionnaire included demographic questions on age, sex, and role with subsequent closed-ended questions based on the first-round results. Participants were presented with a “drag and drop” sorting option for all closed-ended questions, with no mandatory question. Identified components within the Research Priorities were categorised as either a priority to be addressed in the next 2 years, the next 5 years or the next 10 years. Identified components within the Key Resources and Key Processes were categorised as either imperative, important but not essential or not important. Finally, identified components for Outcomes were categorised as essential, useful but not essential or not really useful. The second-round questionnaire was open from the 22nd of November to the 1st of December 2022.

All participants who provided an email address at the end of the second-round questionnaire were sent the link for the third-round (final) questionnaire. Identified components that reached consensus in the second round were not presented again, with all other components presented with a Likert scale. The first section of the questionnaire included an infographic with a summary of the results from the second-round questionnaire (Appendix 2). Demographic questions on age, gender, and role were repeated in this questionnaire. The structure of the closed-ended questions differed in third round, with each component requiring a response. As in the second round, the identified components within the Research Priorities were categorised as in the next 2 years, the next 5 years or the next 10 years, with the addition of a “Not a priority” option. Questions relating to Key Resources, Key Processes and Outcomes were in the same themes as the second round but were presented in Likert format. The third-round survey was open from the 9th to the 20th of Dec 2022.

All questionnaires were initially drafted by one researcher (KAB) and then checked by members of the research team prior to data collection. This ensured there was no conflict of interest as this researcher was based outside of Ireland. Clarifications on the question content validity was made with PHECC representatives prior to each round.

### Analysis

Content analysis was completed on all responses to the first-round questionnaire. Responses were initially coded by one researcher (LH) with codes then collated into broader themes by a second researcher (KAB). This approach ensured no conflict of interest in coding as both researchers are external to the Irish pre-hospital research setting. The themes were checked by the broader research team to ensure a contextual understanding of the results with minor adjustments made as needed.

During the Delphi process no categories were combined, with 70% of the cohort required to select the same category/Likert level, for an item to be deemed as meeting consensus. Graphical representation of results was used to demonstrate consensus level. Demographic data was summarised with percentages and medians and interquartile ranges. Subcategory analysis of all components of the four essential elements was completed by gender and participant role.

### Ethical approval

Ethical approval for this study was obtained from the Monash University Human Research Ethics Committee prior to any participant recruitment (ID: 32147).

## Results

**Fig. 1:**
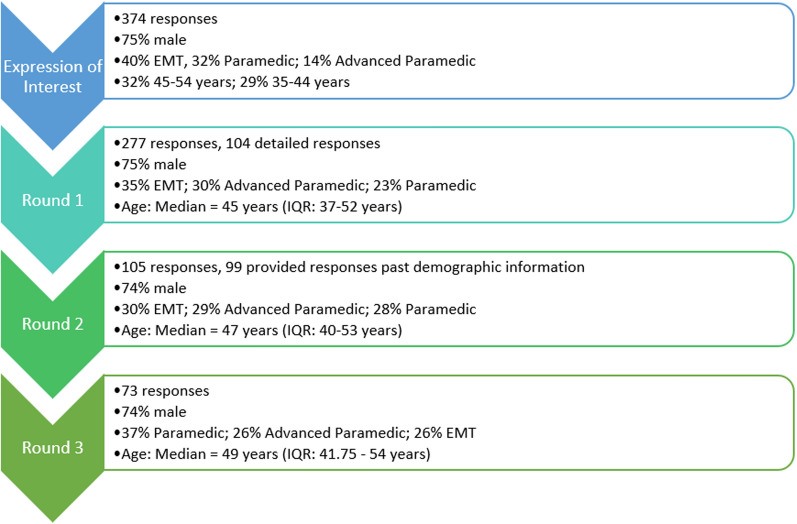
Respondent demographic information

Three-hundred and seventy-four respondents submitted an expression of interest to participate in the project, including a complete email address for future correspondence. Figure 1 shows demographic information of respondents at all stages of the project. These demographic percentages are in line with current PHECC registration data with 72% of paramedicine professionals identifying as male and 29% of registrants falling into both the 46–55 years, and the 36–45 years age groups [18]. Figure 1 also shows response numbers for each round. As a snowballing methodology was used for recruitment it is not possible to calculate a response rate for the initial recruitment. That said as can be seen in the response numbers from Round 2 and 3, 74% of those eligible to complete the final round of the Delphi did so.

**Fig. 2:**
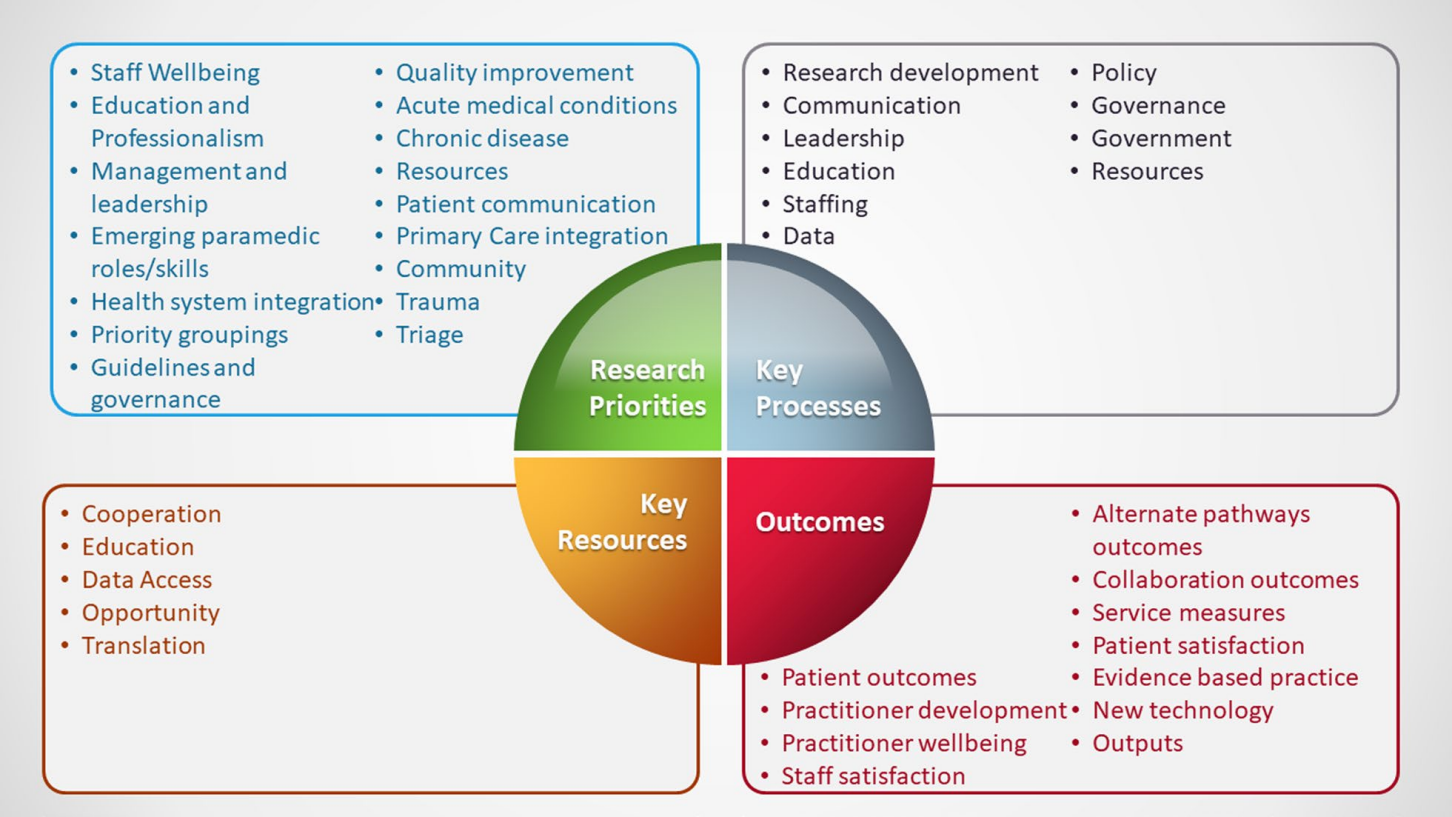
Essential elements and components identified through the Delphi study

Figure 2 shows the components that were identified in the first round for each of the four essential elements of the study.

### Research priorities

Sixteen categories were developed from the first-round open-ended responses in regards to Research Priorities (Fig. 2).

In the second round, 72% of respondents indicated that Staff Wellbeing was a category within the Research Priorities that should be addressed in the next 2 years. Components of the Staff Wellbeing category included: critical incident stress management; ergonomics; first responder wellbeing; human factors; job satisfaction; musculoskeletal injuries; peer support; resilience; salary; and work conditions. As this number was greater than the pre-set consensus number of 70%, this category was deemed to have reached consensus as a priority and was not presented again in the third-round questionnaire.

**Fig. 3:**
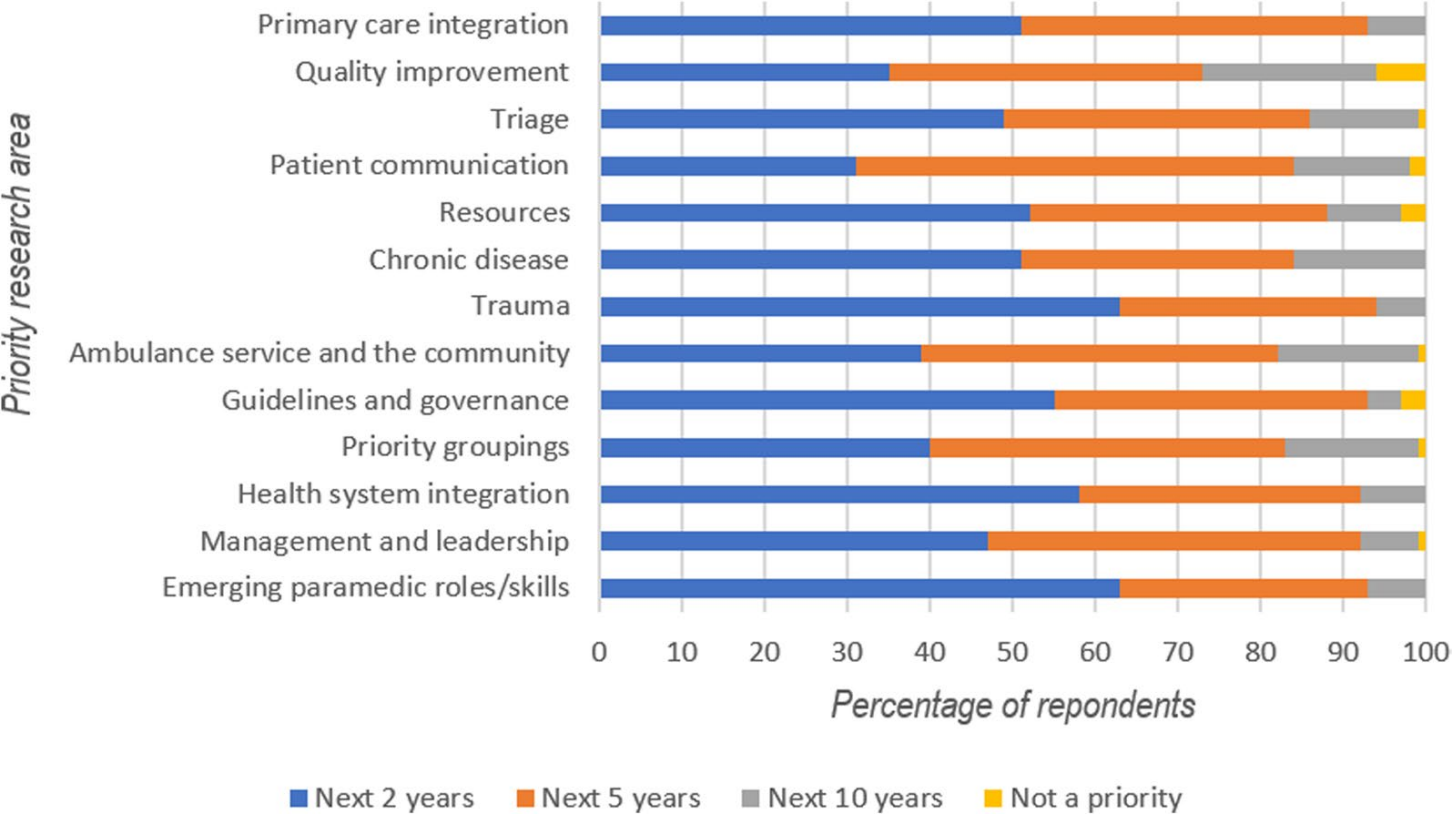
Percentage of respondent breakdown for each time frame for Research Priorities that did not reach consensus

In the third round, 79% of respondents indicated that Education and Professionalism was a Research Priority for the next 2 years. This category included: barriers to research; bridging courses; career progression; EMT education; professional learning and registration; previous education; simulation-based learning; and transition to practice. In addition, 73% of respondents indicated that Acute Medical Conditions (Anti-Emetics, COVID-19, ECGs, fluids, NSTEMI, OHCA, pain relief, sepsis, stroke and vaccination side effects) was also a priority area for the next 2 years. No other priority area reached consensus for any individual time frame. A full breakdown of the Research Priorities that did not reach consensus can be seen in Fig. 3.

In terms of a subgroup analysis for Research Priorities, both genders reached consensus stating that Education and Professionalism was a Research Priority for the next 2 years; however, females did not reach consensus on Acute Medical Conditions being a priority in the next 2 years. Consensus was almost reached for males when indicated that Emerging Paramedic Roles/Skills should be a priority area in the next 2 years (69%); however, this result was not seen for females. It should be noted that the gender distribution between the different roles was not equal with only one female advanced paramedic (AP) included in this cohort and a greater female percentage in the EMT and “Other Roles” category.

The professional role of the participants also led to some differences in responses. When compared to the overall cohort results, Paramedics approached consensus on Acute Medical Conditions (68%) and Emerging Paramedic Roles/Skills (68%) being priority areas in the next 2 years. APs approached consensus on Education and Professionalism (67%) being a priority area in the next 2 years; however, they did reach consensus on Resources and Emerging Paramedic Roles/Skills being priority areas in the next 2 years. Those who indicated that they came from “Other Roles” reached consensus on Health System Integration (70%), Trauma (70%) and Guidelines and Governance (90%) as priorities in the next 2 years.

### Key resources

Ten categories were developed from the first-round open-ended responses with regard to Key Resources (Fig. 2).

**Fig. 4:**
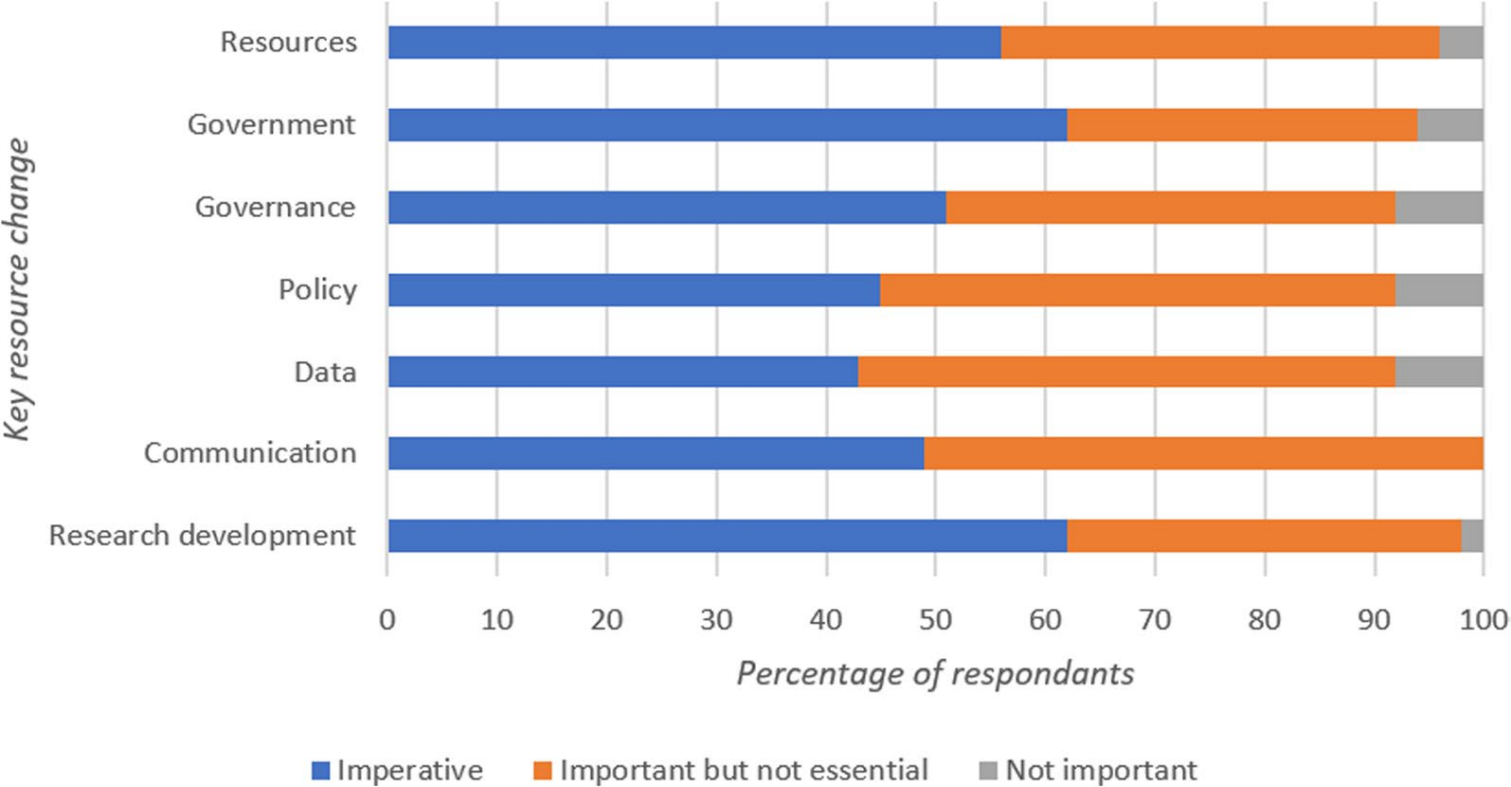
Percentage of respondent breakdown for each importance level that did not reach consensus with regard to Key Resources

No Key Resources reached consensus in the second round of the Delphi study as no category had 70% of the cohort select the same Likert level. All options were re-presented in the third-round questionnaire. In the third round, consensus was reached with participants indicating that there were a number of Key Resources categories that were imperative. This included Education (Access to upskilling/Research skills, Access to university and Continuous professional education; 83%), Staffing (Defined roles, EMT investment, Flexible work arrangements/Time allowance, recruitment of clinical personnel and staff engagement surveys; 78%) and Leadership (Leadership reform, Management buy-in and training, Paramedic representative groups and Transparency from leadership; 70%). No other Key Resources reached consensus for any individual level of importance. A full breakdown of the Key Resources areas that did not reach consensus can be seen in Fig. 4.

In relation to subset analysis for Key Resources by demographic variables, females approached consensus (68%) on the fact that Leadership was a resource change that was imperative to allow the research to occur. Although EMTs did not differ in their consensus items when compared to the full cohort, Paramedics (64%) and those categorised as Other Roles (40%) did not reach consensus for Leadership as an imperative resource change. APs reached consensus for imperative resource changes in Research Development (72%) and Government (72%). Participants categorised as working in “Other Roles” reached consensus for imperative resource changes in the areas of Government (70%) and Resources (70%).

### Key processes

Five categories were developed from the first-round open-ended responses in regards to Key Processes (Fig. 2).

**Fig. 5:**
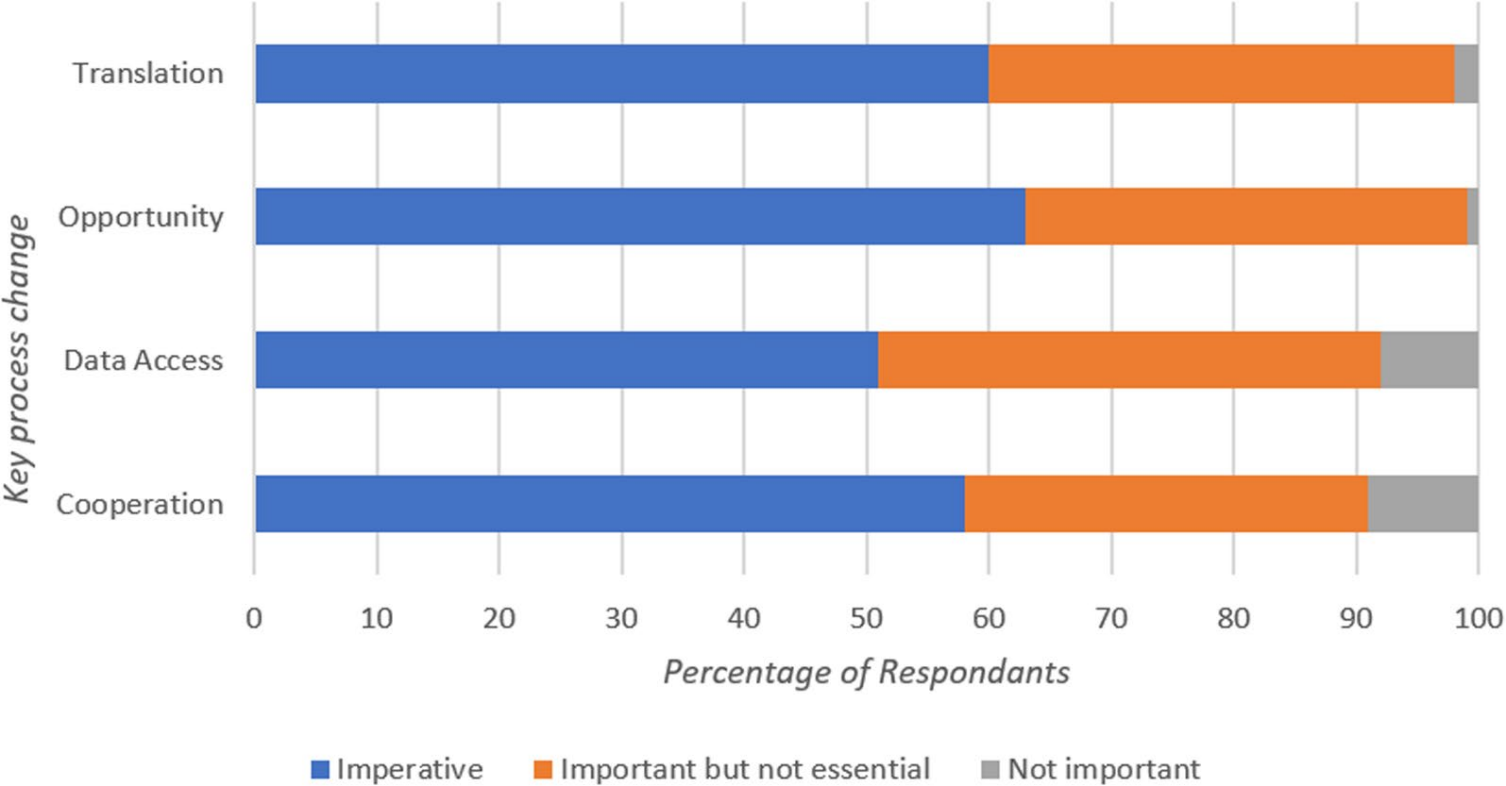
Percentage of respondent breakdown for each importance level that did not reach consensus for Key Processes

In the second-round, 70% of respondents indicated that Education was a Key Processes change that was imperative for research to be completed and thus was deemed to have reached consensus. Components of the Education category included; Education Pathway Restructure, Engagement with Universities, Recruitment of Education Specialists. This Key Processes change was the only option not presented again in third round questionnaire. No other Key Processes reached consensus in the third round at any Likert level. A full breakdown of the Key Processes areas that did not reach consensus can be seen in Fig. 5.

For the subgroup analysis by gender, females reached consensus on the imperative importance of the Key Processes change areas of Cooperation (74%) and Opportunity (74%). In addition, when examined by role, EMTs felt it was imperative to see changes in the area of Opportunity (89%), and APs felt it was imperative to see changes in the areas of Translation (83%) and Cooperation (72%).

### Outcomes

Eleven categories were developed from the first-round open-ended responses in regard to Outcomes for the future strategy (Fig. 2).

**Fig. 6:**
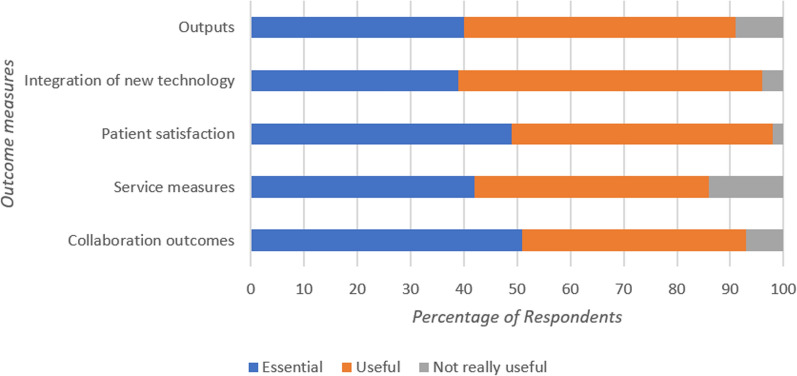
Percentage of respondent breakdown for each importance level that did not reach consensus for Outcomes required in the strategy

None of the Outcomes reached consensus in the second round of the Delphi study as no category had 70% or more of the participants select the same Likert level. All options were re-presented in the third-round questionnaire. In the third round, consensus was reached where participants indicated that certain Outcomes were essential for inclusion in the research strategy. These included Practitioner Wellbeing (mental health of practitioners; 85%), Evidence-based Practice (CPGs with clear evidence base; 78%), Practitioner Development (development of clinical and research skills; 77%), Patient Outcomes (morbidity, mortality and pain management; 75%), Staff Satisfaction (including staff retention; 75%), and Alternate Pathway Outcomes (patients diverted from ED and treated in the community; 71%). A full breakdown of the Outcome measures that did not reach consensus can be seen in Fig. 6.

In subgroup analysis for the Outcomes, consensus differed by gender. Although the male cohort responded similarly to the full cohort, females approached consensus on Alternate Pathway Outcomes, Evidence-based Practice or Staff Satisfaction (all 68%). Females reached consensus on the Outcomes of Integration of technology (74%) as a useful but not essential measure to be included in the strategy. When analysed by Clinical Role, EMTs did not reach consensus on Alternate Pathway Outcomes (53%) being essential and Paramedics did not reach consensus on Patient Outcomes (52%) or Evidence-based Practice (68%) being essential. APs did reach consensus on Service Measures being useful but not essential (83%) and they did not reach consensus on Practitioner Development being essential (61%). Finally, participants categorised as Other Roles did reach consensus for Outputs to be an Outcome measure that is useful but not essential (80%).

**Fig. 7:**
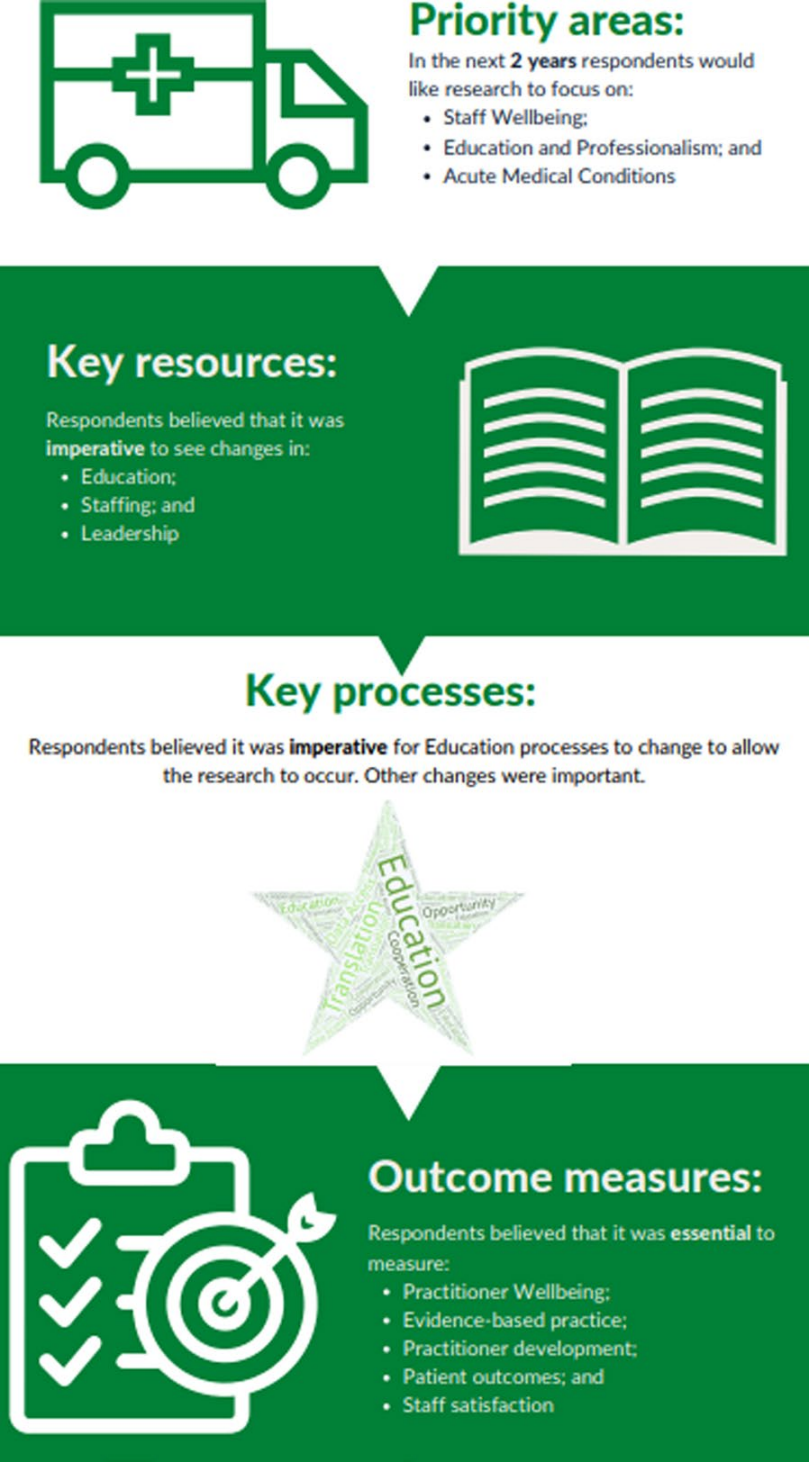
Final research priorities established from the Delphi study

Figure 7 provides an overall summary of the results which was shared with participants after study competition.

## Discussion

In recent years there has been increased recognition of the need for evidence-informed priority setting in healthcare as research is essential to ensure that evidence gaps are translated into actionable knowledge. A total of sixteen Research Priorities in paramedicine were initially identified in this Irish priority setting exercise and the priorities that reached consensus were Staff Wellbeing, Education and Professionalism and Acute Medical Conditions. Participants indicated that these three areas should be a priority for Irish paramedicine research in the next 2 years.

Staff Wellbeing was identified as the most important of the Research Priorities in this study reaching consensus in second round of the Delphi process. In agreement with the findings of this study, a Delphi exercise conducted in the United States in 2022 reported that “Practitioner Wellness” was a priority operational gap in prehospital evidence-based guideline development [13]. Workforce psychological stress and anxiety, in addition to occupational burn-out has also recently been identified as a research priority in a recent Delphi study from Saudi Arabia [14]. COVID-19 has significantly impacted the health and wellbeing of paramedic professionals globally, and is likely to be a main contributing factor to this new finding, considering that wellbeing was not identified as a priority in research priority setting exercises conducted prior to the onset of the pandemic [10, 12]. While all stakeholders in the prehospital and out-of-hospital field must acknowledge the importance of this finding, the onus may be on Service Providers to address some of these issues, particularly those relating to job satisfaction, salary, and work conditions.

Education and Professionalism also reached consensus in this Delphi and was considered a Research Priority for the next 2 years. This finding concurs with a recent Delphi study conducted in Saudi Arabia which highlighted continuous education and training as a prehospital research priority [12]. While paramedic education in Ireland has progressed significantly in recent years with the transition to Higher Education in 2016, this finding suggests that research education still requires development. Service providers and academic institutions need to enhance collaboration with specialist expertise in teaching research skills.

Acute Medical Conditions also achieved consensus as a Research Priority in this study, which agrees with the findings of research priority setting exercises undertaken in the UK in 2009 and 2023 [10, 15]. While management of OHCA and stroke patients featured in both studies, participants in our study also highlighted the need for research into COVID-19 and vaccination side-effects, reflecting the new clinical challenges faced by paramedics in this post pandemic era.

In relation to subgroup analysis for Research Priorities by professional role, APs reached consensus on Emerging Paramedic Roles/Skills and Resources being priority areas in the next 2 years. As APs comprise a higher clinical role for Practitioners on the PHECC Register it would seem logical that this cohort would be particularly interested in emerging roles (including that of Research Paramedics) and the development of new skills and competencies. Participants from “Other Backgrounds” reached consensus on Health System Integration, Trauma, and Guidelines and Governance being priorities area in the next 2 years. These differing priorities likely reflect the diverse roles occupied by these respondents, including those outside of frontline clinical care in the prehospital setting. Although the input of these stakeholders is important as the delivery of urgent care requires collaboration across the health system, the representation of this group was limited in this study. Additional data from these stakeholders that are not paramedic professionals would enhance the understanding behind their differences in priority setting.

Within Key Resources participants initially identified ten resources required to enable research in Ireland. Education, Staffing and Leadership ultimately reached consensus as imperative. Specific components relating to education included access to upskilling, research skills, university education and continuous professional education. Enhancing accessibility to formal Higher Education courses (Certificates, Diplomas, bachelor degrees, masters degrees and Ph.D.’s) and the development of curricula for more informal and flexible researcher training programmes (e.g., research workshops, journal clubs, digital badges) should be prioritised to address these issues. Key Resource changes in relation to Staffing can be directly addressed most effectively by service providers but may require additional Government funding to implement. Some of these specific aspects include: investment and recruitment; defined roles; mechanisms for staff engagement (e.g., surveys); and flexible work arrangements. Protected time for research has previously been identified as a potential facilitator to research in Ireland [2]. Similarly, Service Providers may be best positioned to address resource changes in Leadership, with the support of other stakeholders in the field. While collaboration will continue to be important going forward, empowering paramedics to take ownership and lead research themselves is a vital step for the profession. Action items to drive this include: promoting best practice in research and highlighting quality in research through the publication of research impact case studies; incentivising research and supporting research performance by offering awards; bursaries and scholarships for research excellence; identifying research talent at all career stages and across clinical roles; peer-support; research mentorship; establishing advice clinics; and the appointing of Paramedic Research Champions.

In subgroup analysis on imperative Key Resources both APs and Other Roles reached consensus on the role of Government (e.g., funding, reform). APs also considered Research Development (funding for clinician researchers, data analysts, research assistants and research officers) to be imperative while Other Roles considered Resources (e.g., investment in equipment and infrastructure, library access) as imperative. Enabling the Key Resources changes described here could yield significant progress in building an active research culture in Irish paramedicine.

Regarding Key Processes, a total of five processes required for research were initially identified in this study including: Cooperation; Data Access; Education; Opportunity; and Translation. However, Education was the only Key Process considered imperative which reached consensus in the full cohort. Education is a core pillar of Research Capacity Building, which is defined as a process of developing sustainable abilities and skills enabling individuals and organisations to perform high-quality research [19]. Establishing a national database or register of past and ongoing research projects may support research dissemination, in addition to providing additional opportunities for clinicians to share their research.

In subgroup analysis by gender, females reached consensus on the imperative importance of Cooperation (interorganisational collaboration and stakeholder buy-in) and Opportunity (for participation in research at all clinical levels, involvement in reporting and discussion and reviewing in relation to career progression). When analysed by professional role, EMTs felt it was imperative to see changes in Opportunity while APs felt it was imperative to see changes in Cooperation and Translation (updating of Clinical Practice Guidelines to match current evidence). Cooperation with partners within the care domain has also been identified as a research priority in a recent study based in the Netherlands [11].

A total of eleven Outcomes were initially identified in the first round of the Delphi process with Patient Outcomes, Practitioner Development, Practitioner Wellbeing, Alternate Pathway Outcomes, Evidence-based Practice and Staff Satisfaction being considered imperative and reaching consensus. In relation to Outcomes generally it should be acknowledged that both quantitative and qualitative metrics are important for quality improvement and as indicators of success following research strategy implementation. Patient focused outcome measures (morbidity, mortality and pain management) were also highlighted as being a priority in the UK Delphi study conducted in 2009 by Snooks et al. [10]. Practitioner Development (education in clinical and research skills) and Practitioner Wellbeing (mental health) were again highlighted as being important in this domain and should be captured as outcome measures in research studies. Outcomes relating to Alternative Care Pathways (patients diverted from ED and treated in the community) are considered relevant, which is in agreement with a recent Delphi study that reported mobile care consultation and non-conveyance as a core research theme in the Netherlands [11]. This finding may reflect the current pressures on both the Ambulance Services and EDs in Ireland. It may also relate to the development of new roles in Ireland such as Specialist Paramedics in Community Care and Mental Health, as is the case in other jurisdictions. EBP outcomes including CPGs with a clear evidence base were also a priority as was Staff Satisfaction (including staff retention) which has also been highlighted previously.

In terms of subgroup analysis by gender for Outcomes, there were no differences in imperative measures—however, some differences were observed in relation to measures considered useful but not essential. Analysis by gender demonstrated that females reached consensus on the importance of Integration of technology which was not a feature for the male cohort. When categorised by professional role it was found that APs reached consensus on Service Measures (e.g., response times) while Other Roles reached consensus for Outputs (e.g., numbers of staff involved in research projects, conferences, publications, policies, reports, and curriculum) to be measured in the research strategy.

## Implications of findings

While the findings of this Irish prehospital research priority setting exercise are similar to previously published studies internationally [10–14], some key differences were also observed. The importance of education is frequently noted in existing international paramedicine research strategies [6–8] however, it has not previously been highlighted to the same extent as in Ireland. The requirement of education to enable research in Ireland was evident across all domains of the study; as a Research Priority (Education and Professionalism); a Key Resource (Education); a Key Process (Education); and an Outcome measure (Practitioner Development). This difference may relate to the fact that the transition to higher education of paramedicine in Ireland has been more recent than in other jurisdictions such as Australia, Canada, and the UK, and therefore research training has not yet been fully embedded in paramedic education in the Irish setting. It is apparent from the findings of this study that a renewed emphasis on research education should be targeted by all key stakeholders in the out-of-hospital setting, potentially with a particular focus on collaboration with academic institutions with specialist expertise in research pedagogy.

Staff Wellbeing was identified as the most important Research Priority in this study and Outcomes relating to Practitioner Wellbeing and Staff Satisfaction were also identified as being essential by the participants. Staff Wellbeing as a Research Priority and the need for Outcomes relating to Practitioner Wellbeing had not been highlighted in previous international paramedicine research strategies [10–12]. However, it must be acknowledged that these strategies were all published prior to the onset of COVID-19 and our findings agree with recent Delphi studies in the field from different jurisdictions [11, 14].

Although the findings of this research have provided a strong foundation for the development of a research agenda for paramedicine in Ireland, they may not be generalizable to other healthcare settings and geographical locations. It is recommended that others should engage in their local setting to complete similar work.

## Strengths and limitations

A strength of this study is the diverse international nature of the research team and the inclusive and participatory nature of the Delphi process in involving stakeholders across all clinical and professional roles in Irish paramedicine. A limitation of the work is a lack of representation from other key stakeholders including patients, educators, and service provision managers. Including the perspectives of these groups would provide a more holistic understanding of the research priorities whilst also providing some insight into feasibility. Future research should focus on these stakeholders, potentially with qualitative approaches to augment the findings of this Delphi process.

## Conclusions

This priority setting study for Irish paramedicine research has identified Research Priorities (staff wellbeing, education and professionalism and acute medical conditions), Key Resources (education, staffing and leadership), a Key Process (education) and Outcomes (patient outcomes, practitioner development, practitioner wellbeing, alternate pathway outcomes, EBP and staff satisfaction) to inform the development of a new national strategy in the field. The disseminated findings of this study should inform sustainable funding models to aid the development of paramedicine research in Ireland. A coherent national strategy informed by all key stakeholders can provide a basis for knowledge activities between the academic community and research users based in clinical, educational and policy settings. Future research should focus on identifying the barriers and facilitators to these findings in order to develop specific actions which can enable implementation of this strategy.

### Supplementary Information

Supplementary Material 1

Supplementary Material 2

## Data Availability

No datasets were generated or analysed during the current study.
