# Supplementary figures and images for: Identifying the essential elements to inform the development of a research agenda for Paramedicine in Ireland: a Delphi Study
Source: Health Res Policy Syst. 2024 Aug 9;22:100. doi: 10.1186/s12961-024-01188-6 (PMC11313103; doi:10.1186/s12961-024-01188-6)

Appendix 1


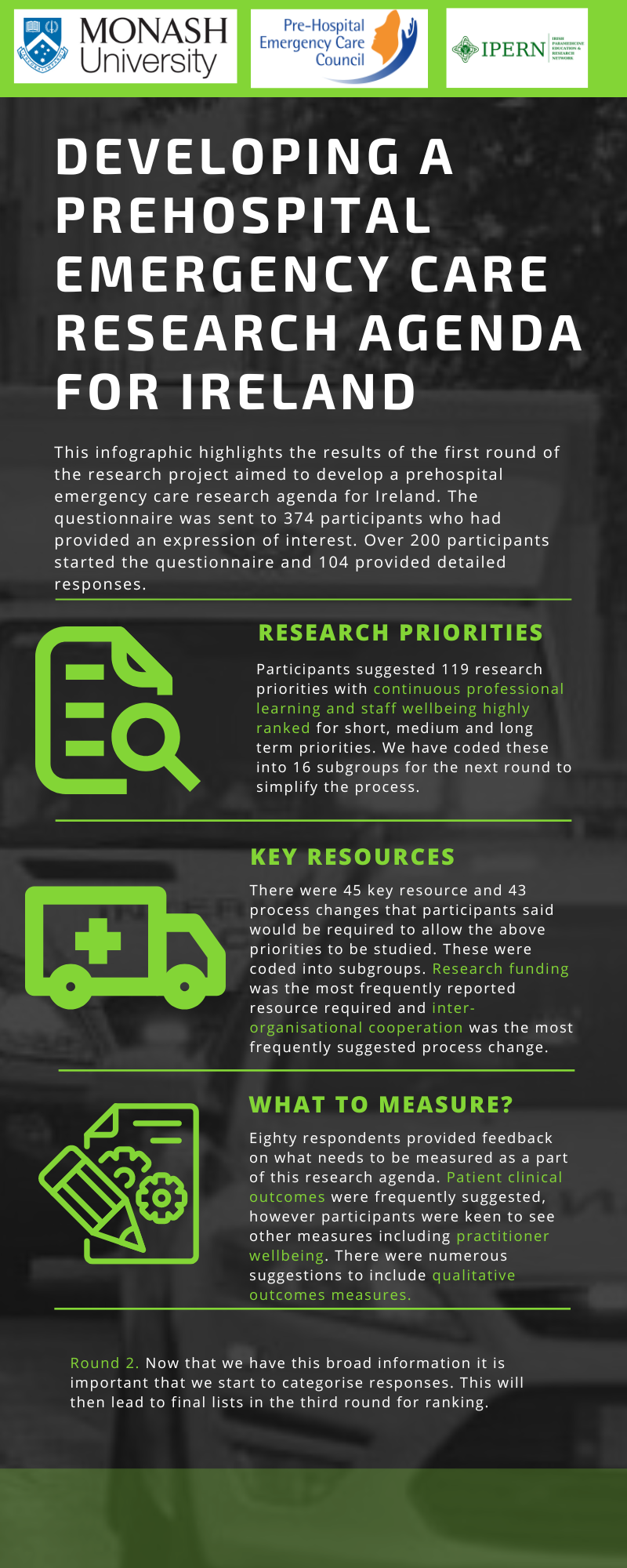

Supplement: Supplementary file 1 — Supplementary Material 1 [file 12961_2024_1188_MOESM1_ESM.docx]

Appendix 2


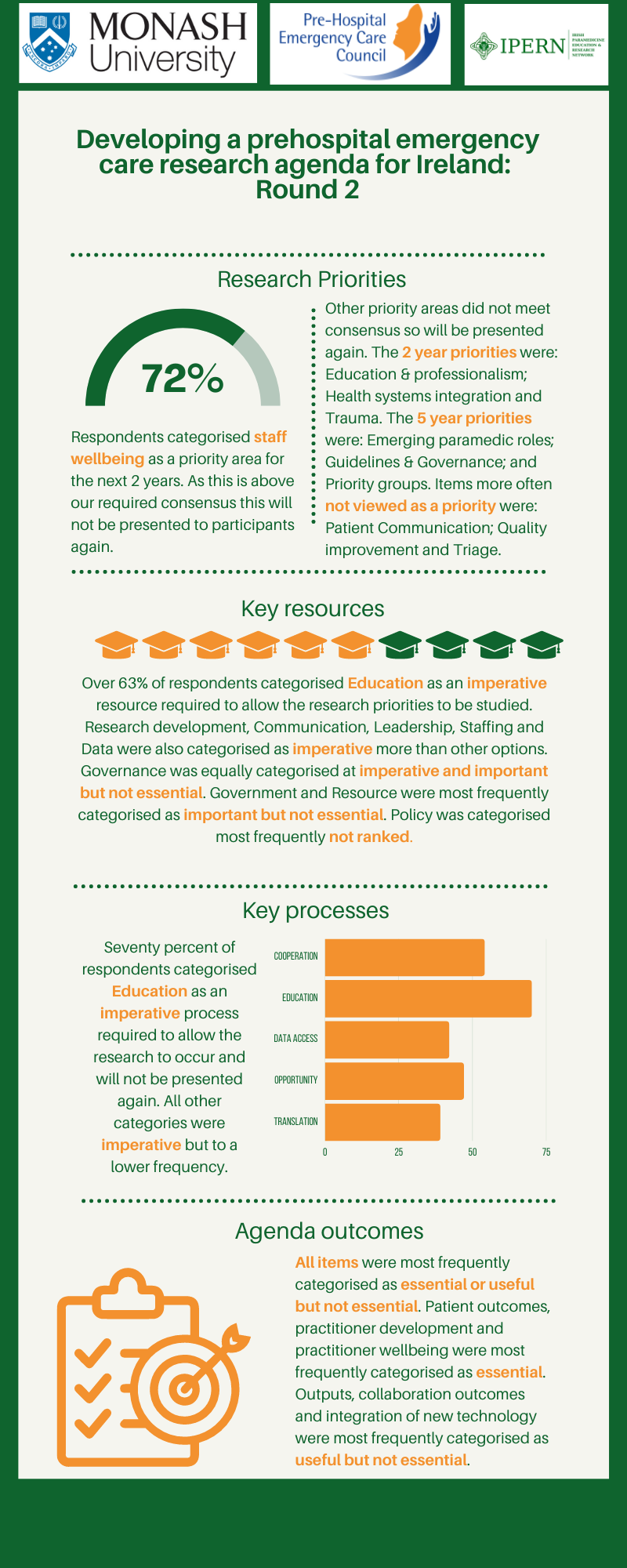

Supplement: Supplementary file 2 — Supplementary Material 2 [file 12961_2024_1188_MOESM2_ESM.docx]
